# A rare case of sino-nasal aneurysmal bone cyst

**DOI:** 10.1016/j.radcr.2022.06.070

**Published:** 2022-07-27

**Authors:** Christian Burgos-Sanchez, Scott Kristenson, Scott Walton, Roy Thomas, Brian Boldt

**Affiliations:** a Department of Radiology, Madigan Army Medical Center, 9040A Jackson Ave, Joint Base Lewis-McChord, WA 98431 USA; b Department of Otolaryngology, Madigan Army Medical Center, 9040A Jackson Ave, Joint Base Lewis-McChord, WA 98431 USA

**Keywords:** Aneurysmal, Bone, Cyst, Sino-nasal, Neuroradiology, MRI

## Abstract

Aneurysmal bone cysts (ABC) are rare in the paranasal sinuses. They are benign expansile multicystic masses containing blood-filled spaces which typically occur in the long bones of pediatric patients. The lesion often produces symptoms due to the compression of adjacent structures or pathological fracture and depends on localization. In this case report, we discuss a 28-year-old female who presented with left-sided headache, left eye proptosis, and diplopia. Radiologic evaluation revealed a left paranasal sinus expansile multicystic mass with internal blood fluid levels displacing and thinning the left medial orbital wall which suggested the diagnosis of ABC. Radiologists should be familiar with and comfortable diagnosing ABC in the head and neck, and be able to differentiate this entity from others, such as telangiectatic osteosarcoma. Biopsy can be challenging since blood products may be the only material identified and may produce tissue that is difficult to interpret or misdiagnosed.

## Introduction

Aneurysmal bone cysts (ABC) are described as benign cystic osseous lesions containing blood-filled spaces separated by connective tissue septa [1]. These lesions infrequently occur in adults, and are rare in the head and neck region with very few cases documented in the paranasal sinuses. ABCs are benign but will present with symptoms owing to their expansile nature. For this reason, treatment will typically be pursued, and it is important for the radiologist to recognize characteristic features to guide accurate diagnosis that will differentiate from other entities such as fibrous dysplasia, metastasis, or telangiectatic osteosarcoma. We present a case of sino-nasal ABC presenting with left-sided headache, left eye proptosis, and diplopia in a 28-year-old otherwise healthy female.

## Case report

A 28-year-old female stationed in Guam as a military dependent presented virtually via tele-health to her physician due to COVID-19 pandemic limitations complaining of a 1-month history of worsening left-sided headaches, vision changes, and left eye proptosis. Due to limited physical exam capabilities virtually, she was treated for migraines with outpatient referral to ophthalmology. She presented 2 weeks later to the emergency department with worsening symptoms where imaging was performed.

**Fig. 1:**
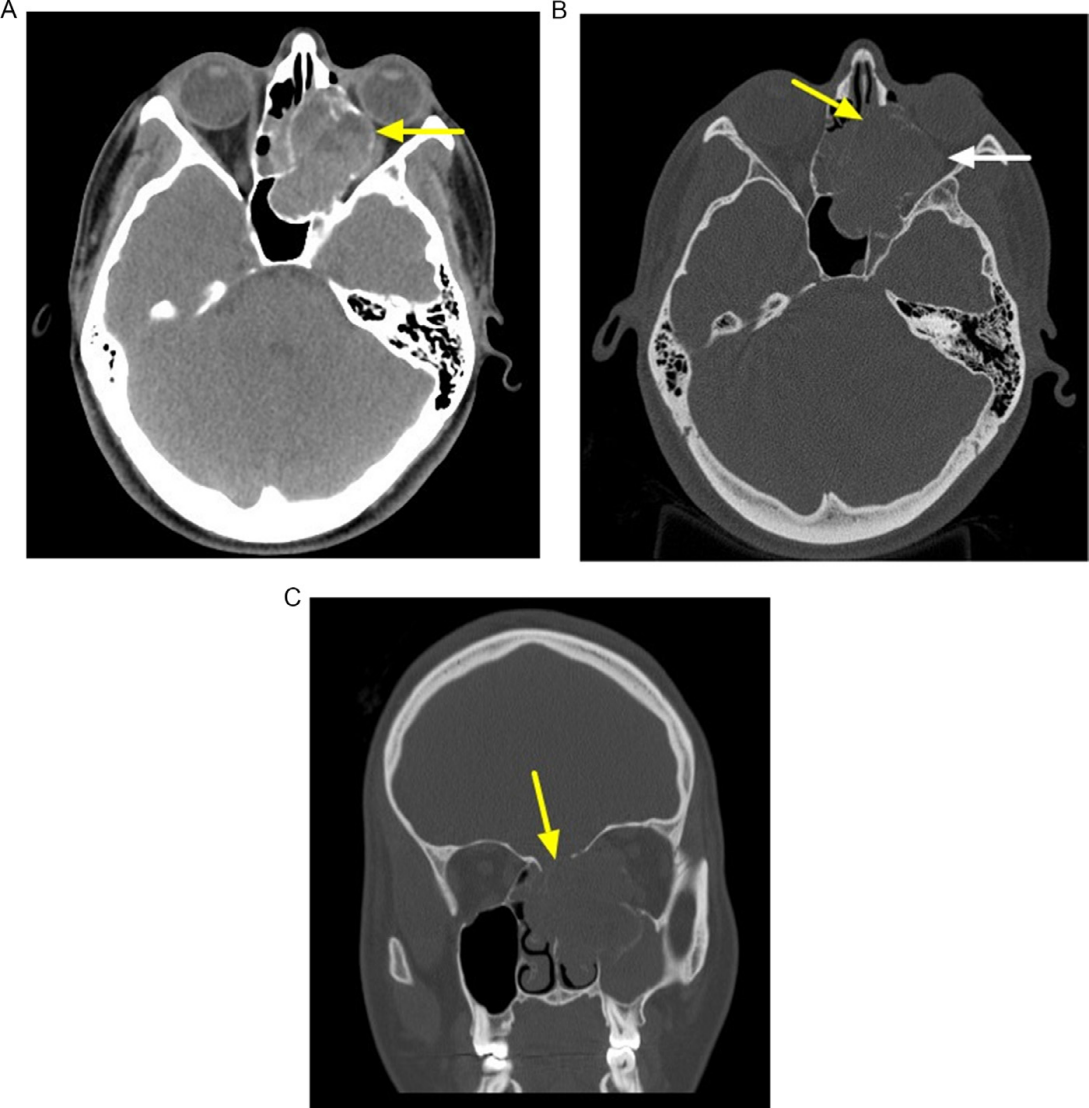
Nonenhanced CT of a paranasal sinus mass. (A) Axial soft tissue window demonstrates an expansile mass arising from the left nasal cavity with internal areas of hyperdensity and several apparent fluid-fluid levels (yellow arrow). Axial (B) and coronal (C) in bone window demonstrates multiple areas of bony expansion and marked thinning/erosion of the medial orbital wall (white arrow) and bony septum (yellow arrow) in Figure B and of the anterior skull base (yellow arrow) in Figure C. (Color version of figure is available online.)

Initial unenhanced CT (Fig. 1) performed of the sinuses demonstrates a large, expansile left paranasal sinus mass with circumferential bony expansion and thinning of the medial orbital wall, bony nasal septum, and anterior skull base with subtle areas of potential hyperattenuating fluid levels. Immediate consultation by ophthalmology revealed a compressive optic neuropathy, restriction of her extraocular muscles, and proptosis. Subsequent nasal endoscopy by otolaryngology revealed a soft, smooth heterogenous mass emanating from the skull base and obstructing a majority of nasal cavity. Biopsy was performed endoscopically during her initial stay.

**Fig. 2:**
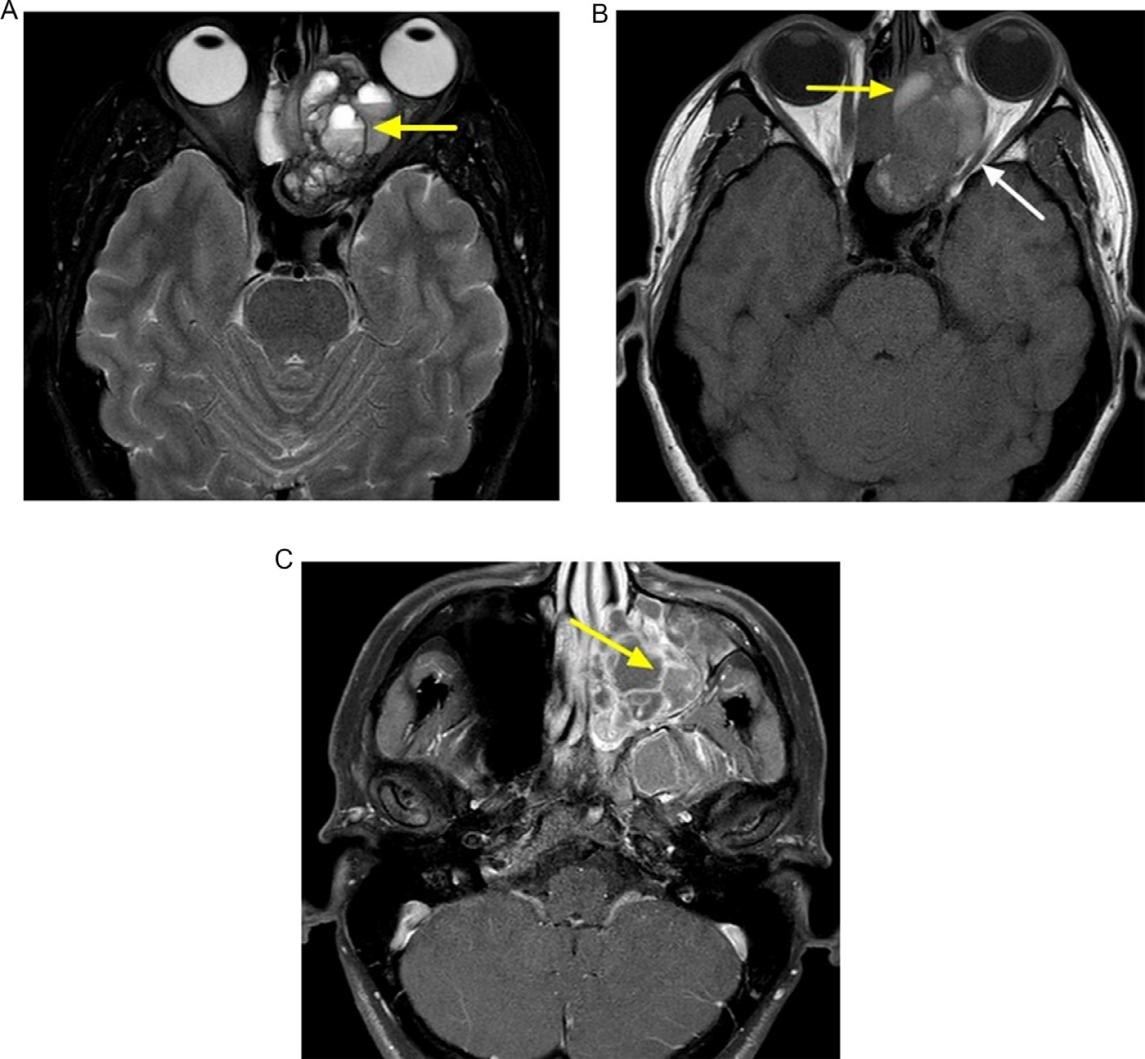
Skull base MRI. (A) Axial T2 confirms multiple cysts with fluid-fluid levels within the nasal cavity mass (yellow arrow). (B) Axial T1 precontrast demonstrates areas of intrinsic T1 hyperintensity consistent with blood products (yellow arrow) and note is made of mass effect on the adjacent left optic nerve (white arrow). (C) Axial T1 postcontrast shows numerous thin, enhancing septations (yellow arrow), without focal enhancing soft tissue component. (Color version of figure is available online.)

Following discharge, and while still awaiting biopsy results, an MRI was obtained (Fig. 2). This study showed a multicystic lesion with multiple areas of intrinsic T1-hyperintense signal compatible with blood products. Postcontrast images demonstrate diffuse, thin septal enhancement without solid mass-like component to suggest a secondary process or other underlying lesion.

The biopsy results revealed the lesion to be consistent with an ABC. Though benign, the mass already proved to be locally destructive in a short time, and given concern for worsening orbital and intracranial complications, surgical removal was recommended. Given the complex surgical requirements necessary for management, the patient was immediately transferred to the United States for specialty care.

Given the destructive appearance of the mass radiographically, and clinical evidence of invasion outside of the sinonasal cavity, a diagnostic surgery with partial tumor removal inferiorly and centrally was performed to allow for reconfirmation of pathology and to exclude a separate underlying process prior to definitive treatment.

Intraoperatively the mass was not highly vascular, but there were areas of hemorrhage with interspersed bony fragments. The peripheral and central portions of the mass had slightly different characteristics so multiple excisional biopsies were taken. The nasal septum was still intact despite concern for erosion preoperatively. The lamina papyracea on the left was completely eroded and the mass was abutting the orbital contents. Despite our concerns for a more sinister process, ABC was again confirmed on all repeat biopsies. The case was discussed at the institution's multidisciplinary tumor board and definitive surgical extirpation was planned. A staged surgery allowing for complete removal of the mass followed by definitive orbital reconstruction to correct the proptosis and extraocular muscle impairments was carried out successfully. Postoperatively, the patient recovered without significant complication and the final pathology again confirmed ABC.

## Discussion

ABCs are relatively rare benign bone lesions, which typically occur in the long bones of pediatric patients. They typically arise in the first 2 decades of life, with a mean age of 11.6 years and a 2:1 female to male predilection [2]. Recent literature; however, demonstrates these predilections may not apply to those that occur in the paranasal sinuses, as in this patient [3]. Its prevalence among all bone tumors is estimated between 1 and 2% [1], and only 2% of ABCs are reported to occur in the head and neck region [4]. ABC also comprises 3% of all bone tumors in the calvarium [5]. When they occur in the head and neck, 66% of ABC preferentially form in the mandible and maxilla, with even rarer occurrence reported in the paranasal sinuses [3].

Presentation of ABC is somewhat variable, and as an expansile lesion will cause symptoms of compressive mass effect and depends on localization. When occurring in the head and neck, the most common presenting symptom is pain and swelling of the affected area [6]. Since the bone architecture is distorted, pathologic fractures have been documented. Those that involve the skull base can cause a number of focal neurologic deficits, to include anosmia, ocular motility palsies, facial weakness, and any number of visual deficits to include diplopia [2]. Of note, nasal discharge and orbital symptoms, particularly proptosis, have been associated with poor outcomes. ABC occurring in the orbit are thought to be more aggressive and resection is difficult. Nasal discharge occurs secondary to obstructive infection which complicates resection and treatment course [1].

ABC were first described in 1942, by Lichtenstein and Jaffe, as “blood-containing osseous tumor” and were said to have similar radiomorphologic characteristics to aneurysms [2]. While the pathogenesis is still relatively not well understood, several theories attribute formation to vascular pathology. Initially, the development of ABC were speculated to be a result of increased venous pressure, which was in turn due to venous thrombosis or an arteriovenous malformation [2]. Bone remodeling then occurs secondary to osteolysis from engorged veins, and the resultant final bony maturation leads to the typically seen findings of bony septations and trabeculation [3]. When ABC occur in isolation, they are classified as primary. Secondary ABC occur when there is previous trauma or prior neoplastic bone change, such as fibrous dysplasia, osteosarcoma, or non-ossifying fibroma [5].

Histopathologic diagnosis is essential in confirming an ABC and ensuring there is no malignancy. Excisional biopsy is preferred over fine needle aspiration and incisional biopsy. Fine needle aspirations tend to only return blood products whereas incisional biopsy is not as accurate and may cause significant hemorrhage [1]. Nonspecific findings such as fibrous proliferation, new bone formation, and blood-filled cysts are often seen in ABC. Key features of ABC are osteoclast-like giant cells and spindle-shaped endothelial cells [1,4] Recently, rearrangement of chromosomal arm 17p and translocation of 16q have been linked to ABC formation [6]. USP6 gene rearrangement has also been recently suggested to aid in differentiation of ABC from telangiectatic osteosarcoma and giant cell granulomas [3].

Radiologic evaluation with CT and MR is essential in providing extent of involvement and suggesting the diagnosis. The presence of an expansile lesion, internal septa, and fluid-fluid levels is characteristic of ABC; however, these findings can also be seen in a variety of other lesions such as chondroblastoma, giant cell tumor, and telangiectatic osteosarcoma. CT is useful in delineating a cortical rim and evaluating the amount of bony remodeling that is affecting adjacent structures [2]. Wide zones of transition and marrow replacement are findings that would be concerning for telangiectatic osteosarcoma. Fluid-fluid levels may be seen on CT, but are readily apparent on fluid sensitive MR sequences.

MRI is, therefore, very useful in suggesting the diagnosis and evaluating for the presence of an underlying lesion/secondary etiology. Fluid-fluid levels are characteristic of ABC and are the result of internal blood products of different ages which will vary in signal intensity and will be most prominent on the fluid sensitive sequences [6]. Surrounding edema within bone and soft tissue will be present on MR as well. A peripheral rim of fibrous tissue that is T1 and T2 hypointense can be used to confirm ABC, and will not be present in telangiectatic osteosarcoma [2]. The presence of an enhancing soft tissue mass or enhancement of the cystic components is not typical of ABC and should suggest another diagnosis such as giant cell tumor or telangiectatic osteosarcoma. Conventional angiography may be helpful to delineate internal vascularity and for preoperative embolization [4].

Although ABC are benign, treatment is usually pursued because of mass effect symptoms and discomfort. Surgical resection is usually pursued and depending on the location a gross total or subtotal resection may be pursued, although recurrence is higher with a subtotal resection [1]. Less invasive options may also be pursued to include sclerotherapy injection, radiation therapy, and embolization [6].

Sinonasal ABC are rare lesions and may present a diagnostic challenge. Radiological features are crucial to predict the diagnosis which should be considered in a patient presenting with an expansile mass with internal fluid-fluid levels. The presence of a soft tissue mass or enhancement of the cystic components suggests a different diagnosis and histopathological diagnosis is necessary to exclude malignant lesions.

## Appendix Supplementary materials

Image, application 1

## References

[1] Smith A.J., Choby G., van Gompel J.J., Link M.J., van Abel K.M. (2021). Aneurysmal bone cysts of the paranasal sinuses: the Mayo clinic experience and review of the literature. Laryngoscope.

[2] Serra A., Gulino A., Di Luca M, Conti A, Maniaci A, Campione G (2017). Sinonasal aneurysmal bone cyst: article review. Acta Med Medit.

[3] McMullen P.D., Bridge J.A., Blair E.A., Yang C.W., Collins J., Cipriani N.A. (2019). Aneurysmal bone cyst of the maxillary sinus with usp6 rearrangement: case report of a rare entity and review of the literature. Head Neck Pathol.

[4] Alghonaim Y., Alsaigh S., Alassiri A.H., Almuntashri M. (2019). Case report: aneurysmal bone cyst arising from the anterior ethmoid sinus. Otolaryngol Case Rep.

[5] Hashemi S.M., Heidarpour M., Eshaghian A., Ansari P., Hashemi M.S., Yaghoobi M. (2015). A Rare Case of Aneurysmal Bone Cyst in the Paranasal Sinus. Iran J Otorhinolaryngol.

[6] Afnan J., Snuderl M., Small J. (2015). Intracranial, intradural aneurysmal bone cyst. Clin Imaging.

