# Supplementary material for: A rare case of sino-nasal aneurysmal bone cyst
Source: Radiol Case Rep. 2022 Jul 27;17(10):3466–9. doi: 10.1016/j.radcr.2022.06.070 (PMC9334923; doi:10.1016/j.radcr.2022.06.070)
Supplement: Supplementary file 1 [file mmc1.docx]

Figure 1: Nonenhanced CT of a paranasal sinus mass. (A) Axial soft tissue window demonstrates an expansile mass arising from the left nasal cavity with internal areas of hyperdensity and several apparent fluid-fluid levels (yellow arrow). Axial (B) And Coronal (C) in bone window demonstrates multiple areas of bony expansion and marked thinning/erosion of the medial orbital wall (white arrow) and bony septum (yellow arrow) in Figure B and of the anterior skull base (yellow arrow) in Figure C.

Figure 2: Skull Base MRI. (A) Axial T2 confirms multiple cysts with fluid-fluid levels within the nasal cavity mass (yellow arrow). (B) Axial T1 Precontrast demonstrates areas of intrinsic T1 hyperintensity consistent with blood products (yellow arrow) and note is made of mass effect on the adjacent left optic nerve (white arrow). (C) Axial T1 Postcontrast shows numerous thin, enhancing septations (yellow arrow), without focal enhancing soft tissue component.
